# Intranasal teeth: a narrative review of clinical features, diagnosis and management

**DOI:** 10.3389/fsurg.2026.1956628

**Published:** 2026-10-22

**Authors:** Sai Hu, Yatian Liu, Xiuli Gao, Haijiao Zhao, Guangtao Liu, Chunchun Chen, Xinyang Jin, Yuxuan Yang, Sicong Ren, Xv Huang

**Affiliations:** 1 Department of Stomatology, The Fourth Affiliated Hospital, School of Medicine, Zhejiang University, Yiwu, China; 2 Department of Otolaryngology-Head and Neck Surgery, The Fourth Affiliated Hospital, School of Medicine, Zhejiang University, Yiwu, China; 3 Center for Regeneration and Aging Medicine, The Fourth Affiliated Hospital of School of Medicine, and International School of Medicine, International Institutes of Medicine, Zhejiang University, Yiwu, China; 4 Department of Stomatology, The First Affiliated Hospital, School of Medicine, Zhejiang University, Hangzhou, China

**Keywords:** ectopic tooth, intranasal tooth, nasal cavity, nasal endoscopy, surgical approach, transnasal surgery

## Abstract

**Background:**

Intranasal teeth are rare ectopic teeth situated wholly or partly within the nasal cavity. Because the evidence is dominated by case reports and terminology and anatomical boundaries vary across reports, diagnostic assessment and management have not been standardised. This review characterises the clinical presentation and synthesises patient-level evidence relevant to imaging, operative planning and multidisciplinary management.

**Methods:**

We conducted a structured narrative review of PubMed, Embase, Web of Science and Scopus, supplemented by citation searching. Reports were eligible when a tooth entered or projected into the nasal cavity; teeth confined to a paranasal sinus without nasal-cavity involvement were excluded from the dataset. The included reports were critically appraised using the appropriate JBI checklist for case reports or case series.

**Results:**

The dataset comprised 139 articles and 170 patient records. Age was reported for 169 patients (median 24.0 years, range 1.67–78), and sex for 169, of whom 107 (63.3%) were male. Supernumerary teeth were reported in 121 records (71.2%), and nasal-floor involvement in 135 (79.4%). The most frequently reported manifestations were nasal obstruction (*n* = 94), rhinorrhoea or purulent discharge (*n* = 73), mass or foreign-body sensation (*n* = 50), epistaxis (*n* = 46), and pain (*n* = 44). Any imaging was documented in 166 records (97.6%), including CT, CBCT or MSCT in 117 (68.8%). Removal or direct extraction was performed in 157 records (92.4%). Critical appraisal identified limitations in adverse-event reporting and post-treatment outcome reporting.

**Conclusions:**

CT or CBCT interpreted with nasal endoscopy and oral examination provides the basis for confirming tooth identity, defining three-dimensional anatomy and selecting operative access. Endoscopic transnasal removal is the predominant published approach, whereas deeply embedded nasal-floor or nasal-maxillary-junction teeth may require oral or combined access. Management should be individualised according to symptoms, anatomical depth, procedural risk and the feasibility of surveillance. The case-based evidence does not support comparative claims between approaches.

## 1 Introduction

An intranasal tooth is a rare ectopic tooth located wholly or partly within the nasal cavity. It may result from aberrant eruption, abnormal development of the tooth germ or acquired displacement. Most are supernumerary, although deciduous and permanent teeth may also migrate into the nose (1–3). The mechanism remains uncertain. Proposed explanations include hyperactivity or splitting of the dental lamina, abnormal development or migration of the tooth germ, maxillofacial trauma or infection, and craniofacial anomalies such as cleft lip and palate (1–3). Intranasal teeth occur in children and adults. Some are asymptomatic and found incidentally on routine dental or sinonasal imaging; symptomatic patients commonly present with unilateral nasal obstruction, rhinorrhoea, recurrent epistaxis, malodour, headache, facial pain or recurrent sinusitis (3–6). These non-specific features can lead to misdiagnosis as a rhinolith, foreign body, osteoma, odontoma or another calcified nasal lesion (4–6). Nasal endoscopy together with computed tomography (CT) or cone-beam computed tomography (CBCT) is central to diagnosis. These modalities can demonstrate a tooth-like radiopaque structure with enamel, dentine and a pulp cavity and define its position, orientation, eruption status and relationship to the nasal floor, septum, inferior turbinate and adjacent dentition. This information guides the choice of surgical approach and anaesthesia (4–6).

Terminology and anatomical scope remain inconsistent. Intranasal tooth, nasal tooth, ectopic nasal tooth and intranasal supernumerary tooth are all used (1–7). Some studies include only supernumerary teeth within the nasal cavity, whereas others also consider deciduous teeth, permanent teeth and teeth displaced by trauma; some combine intranasal teeth with ectopic teeth in the maxillary or other paranasal sinuses (1–3, 6–8). The reported mechanisms and pathological anatomy are similarly heterogeneous, encompassing inverted or ectopically erupted supernumerary teeth, displacement of normal deciduous or permanent teeth, trauma-related migration of tooth germs, abnormalities associated with cleft lip and palate, chronic infection and rhinolith formation (2, 3, 8, 9). Most intranasal teeth lie on the nasal floor, but they may occur along the septum, near the inferior turbinate or at the interface between the nasal cavity and maxilla (3–6). The term rhinolith-associated tooth is best used descriptively for an intranasal tooth with rhinolith formation, rather than as a separate synonym (9). Variation in terminology, aetiology and anatomical boundaries makes denominators, symptom profiles and treatment conclusions difficult to compare across studies.

The wider availability of CT, CBCT and nasal endoscopy has increased detection of asymptomatic and unerupted intranasal teeth and encouraged management tailored to anatomy and presentation (5, 6). A superficially located tooth, or one that has erupted into the nasal cavity, can often be removed directly through the nose or by an endoscopic transnasal approach. A deeply embedded tooth in the nasal floor or maxilla, where transnasal exposure is limited, may require a maxillary vestibular or palatal approach (5, 6, 10). Endoscopy provides direct visualisation and may facilitate dissection while limiting injury to the septum, nasal floor and surrounding mucosa; it is now an important option for most symptomatic cases (10, 11). Removal has commonly been reported in symptomatic patients with nasal obstruction, epistaxis, recurrent infection or threatened adjacent structures.

Despite a growing number of reports, the evidence consists chiefly of case reports and small case series. In 2019, Levin and Sommer reviewed 23 studies comprising 27 patients who underwent endoscopic treatment for ectopic teeth of the nasal cavity or paranasal sinuses, but did not analyse these sites separately (11). Rašić et al. reviewed 50 intranasal supernumerary teeth reported between 1970 and 2020 (7). Kanbe et al. combined 131 published cases with one of their own and examined the location, operative approach and anaesthetic method in 132 patients, proposing a classification based on vertical and horizontal position (6). Their analysis, however, was restricted to intranasal supernumerary teeth without maxillofacial deformity; its applicability to ectopic permanent or deciduous teeth, post-traumatic displacement, cleft lip and palate, and other complex anatomy remains uncertain (6). A 2026 series by Inja et al. included only three patients: two underwent endoscopic removal and one asymptomatic patient was observed (12). Small samples, publication and case-selection bias, inconsistent definitions, incomplete clinical records and short follow-up therefore continue to limit the literature. Epidemiology, aetiological distribution, standardised radiological classification, indications for intervention in asymptomatic patients, and the safety and long-term outcomes of different approaches remain poorly defined.

We therefore undertook a clinical narrative review with a patient-level descriptive synthesis of reports containing extractable data. The primary aim was to characterise the clinical presentation and anatomical distribution of intranasal teeth and to identify imaging, endoscopic and operative factors relevant to otorhinolaryngological surgical decision-making. We additionally examined tooth type, possible aetiological context, management, anaesthesia, follow-up and adverse-event reporting. The synthesis describes the composition and reporting patterns of the published literature; it is not intended to estimate prevalence, compare treatment effects or claim exhaustive capture of all published cases. To minimise anatomical heterogeneity, the main analysis includes only teeth entering the nasal cavity wholly or partly. Teeth confined to the maxillary sinus were excluded.

## 2 Methods

### 2.1 Review design and sources

This study is a narrative review with a structured literature search and patient-level descriptive synthesis of published intranasal tooth cases. The structure and reporting of the review were informed by the six core domains of the Scale for the Assessment of Narrative Review Articles (SANRA) (13). A completed SANRA self-assessment is provided in Supplementary Table S1.

Searches covered PubMed, Embase, Web of Science and Scopus. The search strategy combined subject headings and free-text terms, principally “intranasal tooth”, “intranasal teeth”, “nasal tooth”, “ectopic nasal tooth”, “intranasal supernumerary tooth”, “ectopic tooth” and “supernumerary tooth”, together with anatomical terms including “nasal cavity”, “nasal floor”, “nasal septum” and “inferior turbinate”. Search fields, controlled vocabulary and free-text terms were adapted to the syntax and indexing conventions of each database. Citation tracking of earlier reviews, included reports and citing articles was also undertaken to identify additional potentially eligible reports. Citation tracking did not identify any additional unique eligible reports beyond those captured by the database searches. The database searches were rerun in full on 20 July 2026, which constituted the final search update for this review.

Available bibliographic and institutional resources were used to retrieve potentially eligible full-text reports; however, authors were not systematically contacted and formal interlibrary loan procedures were not undertaken for records that remained inaccessible. Literature identification, deduplication, full-text retrieval and study selection were documented. Detailed search strategies, search dates, flow counts and reasons for full-text exclusion are provided in Supplementary Table S2, and full bibliographic details of the included source articles are provided in Supplementary Table S3. The complete reference list of the 139 articles included in the review is provided in Supplementary File S1.

### 2.2 Scope, operational definition and case selection

The operational definition used in this review was informed by previous clinical reports and reviews describing intranasal teeth and their anatomical distribution (5–7). For the purposes of this review, anatomical eligibility was determined by whether the tooth demonstrably entered or projected into the nasal cavity on the basis of the description, imaging, endoscopic findings or operative findings in the source report. Cases involving both the nasal cavity and maxillary sinus were classified as nasal-maxillary-sinus junction cases and retained when nasal-cavity involvement was clearly demonstrated. Teeth described or shown as being confined entirely to the maxillary sinus, without extension or projection into the nasal cavity, were classified as maxillary-sinus-only cases and excluded. Cases in which the available information was insufficient to determine whether the tooth involved the nasal cavity were excluded as anatomically indeterminate. No minimum percentage of tooth volume within the nasal cavity was required, because no such quantitative threshold was identified in the literature reviewed and could not be applied reproducibly to many older case reports lacking standardised three-dimensional imaging.

Eligible publications were case reports, case series or relevant observational studies of patients with an intranasal tooth. The tooth could be supernumerary, permanent, deciduous or part of the normal dentition. A report had to provide at least one of the following: basic patient information, anatomical location, diagnostic evidence, treatment or clinical outcome. For inclusion in the patient-level descriptive synthesis, reports were required to provide sufficient individual-level clinical information to allow reliable extraction and verification of each patient as a distinct case. Reports presenting multiple patients only in aggregated form, sufficient patient-level extractability was required for final inclusion in the review dataset.

In addition to the maxillary-sinus-only and anatomically indeterminate cases defined above, we excluded teeth confined to the ethmoid, frontal or sphenoid sinus, or to the orbit, without entry or projection into the nasal cavity; roots merely approaching or crossing the nasal floor without entry of the tooth into the cavity; iatrogenically retained roots or fragments, dental implants, restorative material and other dental foreign bodies; odontomas, osteomas, rhinoliths or other calcified lesions without a complete or substantially complete dental structure; reports not based on clinical patient cases; reports with insufficient information to determine anatomical scope; and duplicate publications that added no new patient information.

### 2.3 Case extraction, deduplication and classification

The patient was the principal unit of description, with one record per patient. Patients in multi-case reports were extracted and numbered separately. When a patient had more than one intranasal tooth, tooth count was recorded within the same patient record. Patient-level extraction fields were informed by relevant CARE case-report domains, including patient information, clinical findings and timeline, diagnostic assessment, therapeutic intervention, and follow-up and outcomes. Additional variables specific to intranasal teeth were also extracted (14). CARE was used solely to inform the patient-level data-extraction framework and was not applied as a reporting guideline or critical appraisal tool for the present narrative review. The correspondence between relevant CARE domains and the extracted variables is provided in Supplementary Table S4.

Potential duplicate reports were compared independently by two reviewers using age, sex, country or institution, comorbidity, tooth type, anatomical position, side, treatment and follow-up. Disagreements were resolved through discussion and, when needed, re-examination of the original article. Confirmed duplicate descriptions of the same patient were represented by the most complete report, with other reports used to supplement missing details. When duplication could not be established, records were not merged subjectively and were retained as separate records with their source information. In the final dataset, one pair of patient records could not be definitively classified as duplicate or distinct on the basis of the available source information and was therefore retained as separate records. No formal inter-reviewer agreement statistic, such as Cohen's kappa, was calculated for the screening or duplicate-case assessment stages.

Because older reports varied considerably in content and completeness, unreported information was coded as missing. Absence of reporting was not interpreted as absence of symptoms, complications or recurrence, or as evidence that an examination was not performed (14, 15). Information that was reported but was insufficiently specific or ambiguous for reliable classification was coded as unclear. Tooth type and the aetiology or mechanism proposed in the original report were recorded separately. Tooth type was classified as supernumerary or as belonging to the normal dentition; teeth belonging to the normal dentition were further classified as permanent or deciduous where this could be determined. Cases in which tooth type could not be established were classified as indeterminate. Patient-level coding of the proposed aetiology or mechanism followed the interpretation given in the original report and comprised supernumerary-tooth-related, developmental/congenital, traumatic displacement, iatrogenic displacement, infection/osteomyelitis-related, cyst/tumour-related, genetic/familial and unknown. These categories describe authors' clinical interpretations and do not establish causality.

All reported aetiological factors were retained in the patient-level dataset. Coding was based primarily on the aetiological explanation explicitly proposed by the original authors and, when this was not specified, on the clinical history and anatomical context reported in the source article. Infection, osteomyelitis, cysts, tumours and rhinoliths were also recorded as associated pathological findings or conditions and were not automatically regarded as causes of tooth displacement. They were considered proposed displacement mechanisms only when the original report described a clear temporal sequence or anatomical relationship supporting such an interpretation. When multiple aetiological factors were reported for the same patient, all were retained in the patient-level dataset. For the mutually exclusive tabulation however, each patient was assigned to a single principal aetiological category according to the procedure described above. This principal-category assignment was used solely for descriptive summarisation and did not replace the full set of recorded aetiological factors.

### 2.4 Data items and descriptive synthesis

Extracted variables covered demographic characteristics, clinical history, dental and anatomical features, presentation, diagnostic evidence, management, pathological findings and follow-up outcomes, and included:

- demographic characteristics: age and sex;
- case context: proposed aetiology or mechanism, craniofacial anomaly, trauma, previous surgery and associated pathology;
- dental and anatomical features: tooth type and number, side, specific location, erupted or unerupted status, and orientation;
- presentation: mode of detection, nasal symptoms, oral findings and related complications;
- diagnostic evidence: nasal endoscopy, oral examination, imaging modality and imaging features;
- management: observation or removal, indication, operative approach, anaesthetic method and key procedural techniques; and
- post-treatment findings and outcomes: histopathology, perioperative complications, symptom response, residual or recurrent disease, dentition or craniofacial growth, and duration of follow-up.

For incompletely reported symptom variables, we present the number of symptom present cases, Symptom status assessable cases and the number of cases that Symptom status not assessable. A symptom was considered assessable only when the source explicitly reported its presence or absence, or provided sufficiently clear clinical information for this status to be determined without additional inference. Unreported or insufficiently specified symptoms were classified as not assessable and were not assumed to be absent. Because symptom reporting in case reports is selective, percentages were not calculated for individual symptoms. For examination, imaging, anatomical and pathological variables, reporting completeness was summarised as the number and percentage of patients in whom the relevant information was documented, using the full patient dataset (*N* = 170) as the denominator. Categorical variables with clearly defined denominators are summarised as counts and percentages. Age is reported as the median and range.

The evidence base, comprising predominantly case reports and small case series, is susceptible to selection and publication bias and incomplete reporting (14, 15). Analyses were therefore limited to descriptive statistics, including counts, percentages, medians and ranges. No meta-analysis, significance testing, quality weighting or causal inference was undertaken. We also did not perform comparative effectiveness analyses of observation versus removal, operative approaches or anaesthetic methods.

### 2.5 Critical appraisal

The included reports were critically appraised using the relevant Joanna Briggs Institute (JBI) checklist, with case series assessed using the JBI critical appraisal checklist for case series (15) and case reports using the JBI critical appraisal checklist for case reports (16). Individual domains were rated as Yes, No, Unclear or Not applicable. For case reports, the appraisal covered patient demographics, clinical history and timeline, clinical condition, diagnostic assessment, intervention, post-intervention condition, adverse events and the principal clinical lesson. For case series, the appraisal addressed inclusion criteria, reliable ascertainment of the condition, valid case identification, consecutive and complete inclusion of cases, reporting of demographic and clinical information, outcomes and follow-up, study setting and appropriate analysis. The appraisal was used to characterise methodological and reporting limitations of the predominantly case-based evidence. No report was excluded or statistically weighted on the basis of the appraisal. Study-level ratings are provided in Supplementary Table S5.

## 3 Results

### 3.1 Study selection and case inclusion

The complete database rerun identified 3,807 records: 924 from PubMed, 226 from Embase, 335 from Scopus and 2,322 from Web of Science. After title and abstract screening within each database, 552 potentially relevant records were retained (PubMed, 163; Embase, 104; Scopus, 176; Web of Science, 109). After removal of 303 duplicate bibliographic records, 249 unique reports were sought for full-text retrieval. Full texts could not be retrieved for 104 reports, leaving 145 reports for full-text eligibility assessment. Among the 104 unretrieved records, 57 (54.8%) were published before 2000 and 47 (45.2%) in 2000 or later. 48 (46.2%) were English-language publications, 43 (41.3%) were confirmed as non-English publications, and language information was unavailable or could not be ascertained for the remaining 13 records (12.5%). Bibliographic details of these 104 unretrieved records are provided in Supplementary Table S6.

Six articles were excluded after full-text assessment: one duplicate patient report; one secondary research article; one report that did not meet the operational anatomical definition; one report with insufficient diagnostic confirmation; one non-clinical specimen study; and one 11-case report for which patient-level data could not be reliably extracted and verified. The remaining 139 articles were included in the review. From these articles, 179 candidate patient records were collated. Nine records involving teeth confined to the maxillary sinus without nasal-cavity involvement were excluded, leaving 170 patient records for descriptive synthesis. The complete patient-level dataset is provided in Supplementary Table S7.

### 3.2 Demographic characteristics

The synthesis included 170 patient records, of which age was reported for 169 (99.4%) and sex for 169 (99.4%). The median age was 24.0 years (range, 1.67–78 years). Supernumerary teeth were reported in 121 patient records; among these, age was available for 120, with a median age of 26.5 years (range, 1.67–71 years). Among patients with reported age, 69 (40.8%) were children aged <18 years and 100 (59.2%) were adults aged ≥18 years. Among the 169 patients with reported sex, 107 (63.3%) were male and 62 (36.7%) were female, giving a male-to-female ratio of approximately 1.73:1 (Table 1).

**Table 1:** Demographic characteristics of included patients.

Domain	Measure	*n*	*N*	%
	Clinical cases included	170	170	—
Age	Age reported	169	170	99.4
	Children (<18 years)	69	169	40.8
	Adults (≥18 years)	100	169	59.2
Sex	Sex reported	169	170	99.4
	Male	107	169	63.3
	Female	62	169	36.7

### 3.3 Tooth type and anatomical distribution

Supernumerary teeth were the predominant type (121 patients, 71.2%). Thirty-six patients (21.2%) had permanent teeth, nine (5.3%) deciduous teeth, two (1.2%) teeth belonging to the normal dentition and two (1.2%) teeth of indeterminate type. Eighty-two cases (48.2%) were right-sided and 73 (42.9%) left-sided; eight (4.7%) were bilateral and seven (4.1%) involved the midline or nasal septum. Side was documented in every case (Table 2). The nasal floor was the most common site (135 cases, 79.4%), followed by the nasal septum (14, 8.2%) and the region of the inferior turbinate (14, 8.2%). Four cases (2.4%) involved the nasal-maxillary-sinus junction, one (0.6%) the posterior nasal cavity and two (1.2%) another nasal location (Table 2). Cases explicitly located only in the maxillary sinus were excluded; junctional cases were retained because the tooth also involved the nasal cavity.

**Table 2:** Tooth type, side and anatomical location.

Domain	Category	*n*	*N*	%
Tooth type	Supernumerary	121	170	71.2
	Permanent	36	170	21.2
	Deciduous	9	170	5.3
	Normal dentition	2	170	1.2
	Indeterminate	2	170	1.2
Side	Right	82	170	48.2
	Left	73	170	42.9
	Bilateral	8	170	4.7
	Midline/nasal septum	7	170	4.1
Location	Nasal floor	135	170	79.4
	Nasal septum	14	170	8.2
	Near the inferior turbinate	14	170	8.2
	Nasal-maxillary-sinus junction	4	170	2.4
	Posterior nasal cavity	1	170	0.6
	Other	2	170	1.2

### 3.4 Possible aetiological context

Using the aetiology or mechanism assigned in the patient-level source data, 99 cases (58.2%) were related to a supernumerary tooth, 31 (18.2%) were developmental or congenital, 23 (13.5%) followed traumatic displacement, seven (4.1%) were associated with infection or osteomyelitis, three (1.8%) followed iatrogenic displacement, two (1.2%) had a genetic or familial context, and one (0.6%) was associated with a cyst or tumour, four (2.4%) were of unknown cause (Table 3). These labels reproduce clinical interpretations in the source reports and do not support causal inference.

**Table 3:** Descriptive classification of reported aetiology or mechanism.

Aetiology/mechanism	Cases, *n*
Supernumerary-tooth-related	99
Developmental/congenital	31
Traumatic displacement	23
Infection/osteomyelitis-related	7
Iatrogenic displacement	3
Genetic/familial	2
Cyst/tumour-related	1
Unknown	4

Table 3 presents one principal aetiological/mechanistic category per patient for mutually exclusive descriptive tabulation; these categories should not be interpreted as establishing causality. Tooth type was coded separately. Of the 121 patients classified as having supernumerary teeth (Table 2), 99 were assigned to the principal category of supernumerary-tooth-related aetiology/mechanism, whereas the remaining 22 were assigned to other principal aetiological/mechanistic categories.

### 3.5 Clinical presentation

Nasal obstruction was reported in 94 patients, rhinorrhoea or purulent discharge in 73, a mass or foreign-body sensation in 50, epistaxis in 46, pain in 44, sinusitis or infection in 21, and malodour or crusting in 19 (Table 4).

**Table 4:** Reporting of clinical symptoms in the patient-level dataset.

Symptom	Symptom present, *n*	Symptom status assessable, *N*	Symptom status not assessable, *n*
Nasal obstruction	94	107	63
Rhinorrhoea/purulent discharge	73	79	91
Mass/foreign-body sensation	50	50	120
Epistaxis	46	56	114
Pain	44	47	123
Sinusitis/infection	21	22	148
Malodour/crusting	19	19	151

“Symptom present” denotes explicit reporting of symptom presence. “Symptom status assessable” includes records classifiable as either present or absent. “Symptom status not assessable” denotes unreported or insufficiently described symptom status. Counts describe symptom reporting and should not be interpreted as prevalence estimates.

Reporting completeness varied substantially across individual symptoms. The number of assessable cases ranged from 19 to 107, while the number of cases that were not assessable ranged from 63 to 151. Because case reports preferentially document positive or clinically notable findings, these counts and assessable denominators describe the available published record and should not be interpreted as prevalence estimates.

### 3.6 Reporting of endoscopic, imaging and pathological findings

Imaging was explicitly documented in 166 of 170 patients (97.6%); 117 (68.8%) specifically underwent CT, CBCT or multislice CT. Nasal endoscopy was reported in 81 cases (47.6%) and oral examination in 57 (33.5%). Tooth orientation or axis was reported in 44 cases (25.9%), and histopathology in 45 (26.5%) (Table 5). Thus, imaging was documented for nearly all patients, whereas nasal endoscopy, oral examination, tooth orientation or axis, and histopathological assessment were reported much less consistently.

**Table 5:** Reporting completeness of examination, imaging, anatomical and pathological variables.

Domain	Measure	Reported, *n*	Total *N*	Reported, %	Not reported, *n*
Examination	Nasal endoscopy reported	81	170	47.6	89
Examination	Oral examination reported	57	170	33.5	113
Imaging	CT/CBCT/MSCT	117	170	68.8	53
Imaging	Any imaging reported	166	170	97.6	4
Anatomy	Orientation/axis reported	44	170	25.9	126
Pathology	Histopathology reported	45	170	26.5	125

Percentages indicate the proportion of the full patient dataset (*N* = 170) in which the relevant examination, imaging, anatomical or pathological information was documented. These percentages describe reporting completeness rather than prevalence or diagnostic yield.

CBCT, cone-beam computed tomography; CT, computed tomography; MSCT, multislice computed tomography.

### 3.7 Management, operative approach, anaesthesia and outcomes

Removal or direct extraction of at least one intranasal tooth was performed in 157 of 170 patients (92.4%), while six (3.5%) were managed by observation. Among the remaining seven patients (4.1%), no intervention was documented in four, two experienced spontaneous loss or expulsion of the intranasal tooth, and one underwent surgical repositioning of traumatically displaced permanent maxillary anterior teeth rather than extraction.

Among the 157 patients who underwent removal or direct extraction, 87 (55.4%) underwent endoscopic transnasal removal, 47 (29.9%) a non-endoscopic transnasal approach, 14 (8.9%) an oral vestibular approach, five (3.2%) a combined transnasal-intraoral approach and three (1.9%) other or open approaches. The operative approach was not reported in one patient (0.6%). Among the same 157 patients, general anaesthesia was used in 82 (52.2%), local anaesthesia in 21 (13.4%), and local anaesthesia was converted to general anaesthesia in three (1.9%). Anaesthesia was not reported in 51 patients (32.5%) (Table 6).

**Table 6:** Management, operative approach and anaesthetic method.

Domain	Category	*n*	*N*	%
Management	Removal/extraction	157	170	92.4
Management	Observation	6	170	3.5
Management	Other/non-removal management	7	170	4.1
Approach	Endoscopic transnasal	87	157	55.4
Approach	Non-endoscopic transnasal	47	157	29.9
Approach	Oral vestibular	14	157	8.9
Approach	Combined transnasal-intraoral	5	157	3.2
Approach	Other/open	3	157	1.9
Approach	Not reported	1	157	0.6
Anaesthesia	General	82	157	52.2
Anaesthesia	Local	21	157	13.4
Anaesthesia	Local-to-general conversion/mixed	3	157	1.9
Anaesthesia	Not reported	51	157	32.5

Management percentages were calculated using the full patient cohort (*N* = 170). Operative approach and anaesthesia percentages were calculated among the 157 patients who underwent removal or direct extraction. Other/non-removal management included no documented intervention (*n* = 4), spontaneous loss/expulsion (*n* = 2), and surgical repositioning (*n* = 1).

Follow-up was reported in 100 cases (58.8%) and symptom outcomes were reported for 146 (85.9%). Recurrence or residual disease status was reported in nine patients (5.3%), and dentition or craniofacial growth outcomes in eight (4.7%). Intraoperative event status was assessable in 15 patients, with events reported in six; postoperative event status was assessable in 87, with events reported in four (Table 7). Because perioperative event reporting was incomplete and the assessable denominators were limited, percentages were not calculated for these events. These counts should not be interpreted as complication rates.

**Table 7:** Reporting of follow-up, outcomes and safety.

Outcome/safety item	*n*	*N*	%	Comment
Follow-up mentioned	100	170	58.8	Includes clearly stated follow-up without a quantified duration
Symptom outcome reported	146	170	85.9	
Recurrence/residual disease reported	9	170	5.3	Long-term outcome reporting was sparse
Dentition/growth outcome reported	8	170	4.7	Insufficient long-term evidence in children
Intraoperative event (assessable cases)	6	15	-	Only 15 cases were assessable; not an incidence estimate
Postoperative event (assessable cases)	4	87	-	Only 87 cases were assessable; not an incidence estimate

For intraoperative and postoperative events, counts are presented without percentages because the assessable denominators were limited and should not be interpreted as complication rates.

### 3.8 Critical appraisal

The study-level JBI critical appraisal is presented in Supplementary Table S5. Across the included case reports, diagnostic assessment and intervention were generally described, whereas clinical timelines, explicit adverse-event reporting and post-intervention outcomes were less consistently documented. For case series, ascertainment of the condition and reporting of clinical characteristics were generally adequate, but consecutive and complete case inclusion was frequently unclear. These findings indicate recurrent limitations in reporting completeness and case selection within the underlying literature. No report was excluded or statistically weighted on the basis of the appraisal.

## 4 Discussion

### 4.1 Principal findings

Using a consistent operational definition and excluding maxillary-sinus-only cases, this narrative review delineated the reported clinical presentation of intranasal teeth at the patient level. Published patients were predominantly adults and male, most had supernumerary teeth, and nasal-floor involvement was the most frequently recorded anatomical location. Nasal obstruction, rhinorrhoea, epistaxis, pain and a mass or foreign-body sensation recurred across reports. Most patients underwent removal. Observation was explicitly documented in only six patient records. Separately, explicit absence of complications and long-term follow-up were infrequently documented. The evidence is therefore useful for delineating the clinical presentation and deficiencies in reporting, but not for ranking treatments or setting a firm threshold for intervention.

### 4.2 Demographic characteristics and aetiological context

Male patients accounted for 63.3% of published cases, a distribution similar to earlier reviews of intranasal supernumerary teeth (6, 7). This indicates only that males predominate in the reported literature, not that male sex confers greater risk. Median age was 24.0 years in the overall dataset. Among the 121 patients with supernumerary teeth, age was available for 120; the median age in these 120 patients was 26.5 years (range 1.67–71 years), substantially higher than the median age of 10 years reported by Kanbe et al. in their review of intranasal supernumerary teeth (6). This difference should be interpreted in the context of differences in eligibility criteria and case composition between the two reviews.

Supernumerary teeth comprised 71.2% of patients. Proposed mechanisms for the development and ectopic eruption of supernumerary teeth—including dental-lamina hyperactivity, tooth-germ splitting and abnormal migration—remain largely conjectural (1–3, 6). Tooth type and aetiological context were coded separately. Among the 121 patients with supernumerary teeth, 99 were classified as supernumerary-tooth-related in the aetiology/mechanism dataset, while 22 were assigned to other categories. Other reported contexts included developmental or congenital factors, traumatic displacement, infection or osteomyelitis, previous procedures, genetic or familial factors, and cyst- or tumour-related conditions (Table 3). Original reports document intranasal displacement involving both deciduous and permanent teeth after trauma (17, 18). In the case reported by Dokania et al., the history of a missing deciduous incisor after trauma, oral examination and the morphology of the extracted tooth supported identification of the intranasal tooth as part of the normal dentition (17). Together with reports involving cleft lip and palate (19), these cases support recording dental identity, trauma history, previous procedures and developmental anomalies separately rather than assuming that every intranasal tooth is supernumerary.

### 4.3 Anatomical distribution and presentation

Nasal-floor involvement was recorded in 79.4% of patients. This distribution is consistent with earlier series and reviews (4–7) and Lee's series of 13 patients, in which all intranasal teeth were located on the nasal floor, although their mucosal covering and association with rhinolith formation varied (20). Left and right sides were similarly represented in the present dataset. Bilateral, midline and septal locations were less frequently reported (21–24). Clinical appearance may vary with anatomical location. Mohebbi et al. described an intraseptal tooth presenting with septal deviation and nasal obstruction, without a visible white intranasal mass (24).

Nasal obstruction and rhinorrhoea or purulent discharge were the most frequently recorded manifestations in the present dataset; epistaxis, pain and a mass or foreign-body sensation were also repeatedly reported. Lin et al.'s three cases included nasal obstruction accompanied by epistaxis or purulent rhinorrhoea (25). Al Dhafeeri et al. described recurrent unilateral epistaxis in a patient whose intranasal tooth contacted the anterior nasal septum; no further episodes were reported at the three-month postoperative review (26). Such reports illustrate the range of clinical presentations but do not establish symptom prevalence. Reporting of less commonly described symptoms was incomplete; malodour or crusting was documented in 19 patients, whereas its presence or absence could not be assessed in 151 patients.

### 4.4 Diagnosis, imaging assessment and differential diagnosis

Imaging was documented in 166 of 170 patients (97.6%), including CT, CBCT or multislice CT in 117 (68.8%). In Chen et al.'s report, tooth-equivalent attenuation and a central cavity corresponding to the pulp space supported the diagnosis, while CT also demonstrated surrounding soft tissue and the relationship to the nasal floor (4). Albert and Mupparapu used multiplanar CBCT reconstructions to show an inverted mesiodens extending across the nasal floor into the nasal septum (27). These reports illustrate how cross-sectional imaging can establish dental morphology and define orientation, depth and relationships to adjacent structures. However, the appearance is not invariably diagnostic: in Mohebbi et al.'s intraseptal case, close proximity to bone made the tooth difficult to distinguish on preoperative imaging (24). Imaging should therefore be interpreted together with nasal and oral examination when distinguishing an intranasal tooth from a rhinolith, foreign body, osteoma, odontoma or another calcified lesion (4, 9, 17, 24).

Nasal endoscopy was described in only 47.6% of patients, oral examination in 33.5%, and tooth orientation or axis in 25.9%. Kanbe et al. proposed selecting operative access and anaesthesia according to vertical and horizontal impaction (6), but orientation/axis could be reconstructed in only about one quarter of our cases. Moreover, their framework was derived mainly from intranasal supernumerary teeth without maxillofacial deformity. Current evidence is therefore insufficient to establish its generalisability to ectopic permanent or deciduous teeth, traumatic displacement, cleft lip and palate, or nasal-maxillary-sinus junction cases. Future reports should document crown-root orientation, depth of impaction, mucosal exposure and three-dimensional relationships to critical structures.

### 4.5 Management and implications for clinical decisions

Removal or direct extraction was reported in 157 of 170 patients (92.4%), and endoscopic transnasal removal was the most frequently documented approach. Lee's series provides relatively detailed outcome information: all 13 patients underwent endoscopic extraction, with uneventful recovery and reported follow-up ranging from six months to 14 years and five months (20). Accorona et al. also described endoscopic removal of a tooth arising from the nasal floor, with symptom resolution and endoscopic evidence of healing at one month (28). These observations describe outcomes in selected patients and do not establish the comparative superiority of endoscopic treatment.

Oral or combined access may be required when tooth position, bony attachment or intraoperative difficulty limits transnasal removal. Costa et al. described a modified maxillary vestibular approach (29), while Gormley et al. reported successful intraoral removal after unsuccessful nasoendoscopic attempts (30). Sukegawa et al. described combined oral and nasal access to retrieve a retained root after fracture of an intranasal supernumerary tooth during extraction (31). These cases illustrate the relevance of three-dimensional position, impaction depth, root morphology, mucosal exposure and adjacent anatomy to operative planning. Comparative data remain insufficient to rank the available approaches.

Observation was explicitly documented in six patients (3.5%), with limited information on long-term outcomes. Albert and Mupparapu described an asymptomatic patient with an inverted mesiodens involving the nasal floor and septum, for whom continued monitoring was planned (27). In Inja et al.'s series, a right-sided intranasal tooth was left in place because it caused no ipsilateral symptoms and did not interfere with septoplasty for a symptomatic left-sided septal spur (12). Conversely, Lee's series included five asymptomatic patients whose intranasal teeth were removed (20). These reports document different management choices but do not establish whether observation or removal is preferable for an asymptomatic tooth. Decisions may take into account whether symptoms are attributable to the tooth, associated pathology, anatomical position, anticipated procedural morbidity, age and dental development, and the feasibility of follow-up.

Anaesthetic practice varied and was incompletely reported, precluding comparison between local and general anaesthesia. Post-treatment follow-up should document symptom resolution, healing, residual or recurrent calcified material and effects on adjacent dentition; longer-term follow-up is particularly relevant in children.

### 4.6 Potential multidisciplinary considerations for diagnosis and management

Based on the patterns identified in the published literature, evaluation of a suspected intranasal tooth may involve confirmation that the calcified nasal lesion is dental in origin. Nasal endoscopy may help document mucosal exposure, granulation tissue, discharge, bleeding and its relationship to adjacent nasal structures, while oral examination may provide information on missing or delayed teeth, palatal or vestibular swelling, trauma and previous surgery. CT or CBCT can be used to delineate three-dimensional anatomy and may assist with management planning. Otorhinolaryngological assessment may be particularly relevant when intranasal exposure or a transnasal approach is being considered, whereas dental or oral and maxillofacial assessment may assist in evaluating tooth identity, adjacent roots, occlusion, developmental anomalies and the feasibility of oral access. Management decisions may take into account symptoms, associated pathology, anatomical characteristics, operative feasibility and the possibility of observation in selected cases. These considerations are derived from the available case-based literature and are intended to provide a conceptual summary.

### 4.7 Limitations and research priorities

This review has several limitations. First, the evidence base consists of case reports and small case series. The JBI critical appraisal showed that, among the case reports, reporting of clinical timelines and post-treatment outcomes was incomplete in some reports, while adverse events were frequently unclear or not explicitly reported. In several case series, consecutive and complete case inclusion was also unclear. These findings characterise limitations of the available evidence. Second, full texts could not be retrieved for 104 of the 249 candidate records. As detailed in the Results, many of these records were older or non-English publications. Their inaccessibility limited the completeness of the evidence base and prevented assessment of whether their inclusion would have altered the descriptive findings. Third, as a narrative review, the study was not conducted under a preregistered systematic-review protocol and cannot claim exhaustive retrieval of all published cases. Although screening and duplicate-case assessment were performed independently by two reviewers and disagreements were resolved through discussion, inter-reviewer agreement was not formally quantified. One pair of patient records could not be definitively classified as duplicate or distinct; therefore, the dataset of 170 patient records may correspond to 169–170 unique individuals. Fourth, definitions of intranasal and paranasal-sinus teeth varied across reports, and older publications frequently lacked sufficient textual or imaging detail to determine anatomical boundaries with certainty. Reporting of negative symptoms, tooth orientation, endoscopic findings, treatment indications, adverse events and long-term outcomes was also inconsistent, limiting stratified analyses and meaningful comparisons between management approaches.

Future reports should adopt a consistent anatomical case definition and distinguish clearly between patient-level and tooth-level data. At a minimum, reports should include age, sex, tooth type and number, trauma and surgical history, craniofacial anomalies, both positive and negative symptoms, nasal endoscopic and oral findings, three-dimensional CT or CBCT location, crown-root orientation, indication for treatment, operative approach, anaesthesia, perioperative events and long-term follow-up. Multicentre registries or standardised case series may reduce duplicate reporting and missing data and provide more reliable evidence regarding intervention thresholds in asymptomatic patients, the applicability of classification systems and the comparative safety of surgical approaches.

## 5 Conclusions

Intranasal teeth are rare and heterogeneous in aetiology, tooth type and anatomy. The final descriptive synthesis included 170 patient records from 139 articles. Published cases were predominantly adults, male, supernumerary and located on the nasal floor; nasal obstruction, rhinorrhoea, epistaxis, pain and a nasal mass or foreign-body sensation were repeatedly recorded. CT or CBCT, interpreted with nasal endoscopy and oral examination, can establish tooth identity, three-dimensional position and anatomical relationships. The decision to remove the tooth and the choice of operative access should be individualised according to symptoms, anatomical characteristics, procedural risk and the feasibility of surveillance. Current case-based evidence cannot estimate prevalence or determine the comparative effectiveness of management strategies.
