# Supplementary material for: Intranasal teeth: a narrative review of clinical features, diagnosis and management
Source: Front Surg. 2026 Oct 22;13:1956628. doi: 10.3389/fsurg.2026.1956628 (PMC13737578; doi:10.3389/fsurg.2026.1956628)
Supplement: Supplementary file 1 [file Supplementaryfile1.docx]

Intranasal Teeth References

Bibliography of 139 included articles

Entries follow Supplementary Table S1. Reference numbers and NT identifiers are mapped in the appendix.

The blue markers indicate where each reference is cited in the main text of the article.

1. Takahashi M, Hirano T, Nakayama A, Ogawa A, Abe R, Irié T, et al. A case of a rhinolith associated with an inverted impacted supernumerary tooth. J Oral Maxillofac Surg Med Pathol. (2026) 38(3):522-529. doi: 10.1016/j.ajoms.2025.10.011

2. Uma E, Panicker K, Tyagi M. Conservative management of intranasal tooth. Br Dent J. (2026) 240(3):132-133. doi: 10.1038/s41415-026-9586-x

3. Kanbe T, Musha A, Ogawa M, Yokoo S. Criteria for Treatment of Intranasal Supernumerary Teeth: A Comprehensive Literature Analysis. Ear Nose Throat J. (2026) 105(3):NP216-NP225. doi: 10.1177/01455613231186953

4. Jihad A, Wafae L, Hiba T, Zainab EZ, Adil ET. Ectopic nasal tooth: A clinical case report. Radiol Case Rep. (2026) 21(5):2242-2245. doi: 10.1016/j.radcr.2026.02.002

5. Cerantula C, Guimarães ÂM, Pulino B, Guerra RC, Garcia Júnior IR, Scariot R. Ectopic Supernumerary Tooth Displacement into Nasal Cavity: Rare Presentation in Cleft Lip and Palate-Case Report and Literature Review. Cleft Palate Craniofac J. (2026) 63(8):2604-2609. doi: 10.1177/10556656251384610

6. Thakkar H. Ectopic Tooth Eruption from Inferior Turbinate in Nose with Rhinolith. Indian J Otolaryngol Head Neck Surg. (2026) 78(1):415-418. doi: 10.1007/s12070-025-06050-x

7. Kunwar B, Ghimire A, Shah B, Paudel B, Bhandari B, Sharma A, et al. Intranasal ectopic tooth: An unusual diagnostic finding with review of literature. Radiol Case Rep. (2026) 21(10):4230-4235. doi: 10.1016/j.radcr.2026.06.051

8. Inja RR, Pushpa J, Varghese L, Cherian LM, Kurien R. Intranasal Supernumerary Teeth-Case Series and Review of Literature. Indian J Otolaryngol Head Neck Surg. (2026) 78(5):2788-2793. doi: 10.1007/s12070-026-06492-x

9. Nanjo K, Kagoya R, Hinata M, Koda K, Horikiri K, Nishijima H, et al. A Case of Sinonasal Actinomycosis Associated With an Odontogenic Cyst of Ectopic Dental Origin. Cureus. (2025) 17(7):e88209. doi: 10.7759/cureus.88209

10. Edora BDE, Chua RU. A Case Report on Ectopic Intranasal Lateral Incisor Presenting as Chronic Rhinosinusitis in a 25-Year-Old Woman. Philipp J Otolaryngol Head Neck Surg. (2025) 40(2):39-42. doi: 10.32412/pjohns.v40i2.891

11. Cheng YT, Chang KM. Ectopic Tooth Arising from the Nasal Floor. Ear Nose Throat J. (2025). [Epub ahead of print]. doi: 10.1177/01455613251374790

12. Liang W. Ectopic Tooth in the Nasal Cavity Following Maxillary Ameloblastoma Resection. J Coll Physicians Surg Pak. (2025) 35(12):1639-1640. doi: 10.29271/jcpsp.2025.12.1639

13. Grün P, Turhani F, Grün AS, Müßig F, Gutwald R, Turhani D. Maxillary impacted mesiodentes in unusual positions: Two case reports. Int J Surg Case Rep. (2025) 134:111798. doi: 10.1016/j.ijscr.2025.111798

14. Ozcan Kucuk A, Yolcu U. Simultaneous Intranasal Tooth Extraction Using LeFort I Osteotomy in Patient Undergoing Orthognathic Surgery. J Maxillofac Oral Surg. (2025). [Epub ahead of print]. doi: 10.1007/s12663-025-02827-3

15. Umezawa H, Baba J, Aoki N, Yamamoto K. Supernumerary intranasal floating tooth: clinical and literature review. BMJ Case Rep. (2025) 18(6):e260818. doi: 10.1136/bcr-2024-260818

16. Sukegawa S, Samukawa Y, Akiyama K, Nakai Y, Miyazaki R, Miyake M. Collaborative supernumerary nasal tooth extraction by nasal and intraoral approaches. Oral Sci Int. (2024) 21(1):150-154. doi: 10.1002/osi2.1190

17. Bhaduri S, Radder K, Desai AK. Ectopic Maxillary Incisor in the Nasal Cavity: A Case Report and Literature Review. J Maxillofac Oral Surg. (2024). [Epub ahead of print]. doi: 10.1007/s12663-024-02278-2

18. Iacomino E, Fratini C, Zoccali F, Cambria F, Laudani M, Eibenstein A, et al. Endoscopic Surgical Approach for a Mesiodens in the Nasal Cavity: A Rare Phenomenon. Reports (MDPI). (2024) 7(2):46. doi: 10.3390/reports7020046

19. Velayutham P, Vaithilingam Y, Arumugam B, Davis P. Bilateral Supernumerary Teeth Presenting as Nasal Masses and Recurrent Rhinitis: An Interesting Case Report. Indian J Otolaryngol Head Neck Surg. (2023) 75(Suppl 1):1021-1023. doi: 10.1007/s12070-023-03586-8

20. Tsukor SV, Arkhandeev AV, Alaverdov VP. Clinical case: ectopia of supernumerary tooth in the nasal cavity. Clinical Dentistry (Russia). (2023) 26(2):174-176. doi: 10.37988/1811-153x_2023_2_174

21. Zhang H, Tao M, Bai J, Du S. Ectopic nasal tooth mimicking recurrent rhinolith in a young woman: A diagnostic challenge for the otolaryngologist. Asian J Surg. (2023) 46(8):3120-3121. doi: 10.1016/j.asjsur.2023.02.076

22. Rasic M, Mamic M, Biocic J, Luksic I. Intranasal supernumerary tooth: two case reports and review of the literature. Acta Stomatol Croat. (2023) 57(4):395-400. doi: 10.15644/asc57/4/9

23. Elgamri AI, Siddig OEH, Jobran AWM. Supernumerary intranasal tooth: case report and review of the literature. J Surg Case Rep. (2023) 2023(9):rjad537. doi: 10.1093/jscr/rjad537

24. Hong CY. Ectopic nasal tooth. Med J Malaysia. (1976) 30(3):239-240.

25. Raubaite R, Rakauskaite A, Sukyte-Raube D, Zaleckas L, Rauba D. An Intranasal Ectopic Tooth in an Adult. Cureus. (2022) 14(4):e24410. doi: 10.7759/cureus.24410

26. Noleto JW, de Abreu NMR, Dos Santos Vicente KM, da Silva AVNA, Seixas DR, de Figueiredo LS. Intrusive luxation of permanent upper central incisor into the nasal cavity: A case report. Dent Traumatol. (2022) 38(2):160-164. doi: 10.1111/edt.12727

27. Ferenc T, Zovak G, Vidjak V. Inverted supernumerary nasal tooth: a case report and literature review. Med Jad. (2022) 52(4):303-306. doi: 10.57140/mj.52.4.8

28. Priya M, Doomra S, Angral S, Malhotra M, Bhardwaj A, Varshney S, et al. Supernumerary Tooth: Endoscopic Removal for a Rare Cause of Recurrent Epistaxis. Indian J Otolaryngol Head Neck Surg. (2022) 74(Suppl 2):1143-1146. doi: 10.1007/s12070-020-02221-0

29. Zaheer K, Joshi N, Breen M, Chow V. Surgical Removal of a Canine Displaced in the Floor of the Nasal Cavity: Case Report and Review of the Literature. Indian J Otolaryngol Head Neck Surg. (2022) 74(Suppl 2):984-986. doi: 10.1007/s12070-020-02067-6

30. Anand R, Kieu A, Arulraj E, Ilangovan G, Narmada DA, AshwathNarayana G, et al. A Rare Case of an Intra-nasal Ectopic Tooth in a Young Woman. Cureus. (2021) 13(11):e19370. doi: 10.7759/cureus.19370

31. Ong HY, Ng JJ, Ong HJ, Wong SJ, Gopalan S. A Rhinolith Turning Out to Be an Intranasal Tooth. Cureus. (2021) 13(2):e13616. doi: 10.7759/cureus.13616

32. de Souza Santos AM, de Lima-Neto TJ, Mendes BC, Hochuli Vieira E, Sverzut AT, Luna AHB. Algorithm for Treatment of Supernumerary Nasal Tooth. J Craniofac Surg. (2021) 32(1):e38-e41. doi: 10.1097/SCS.0000000000006868

33. Hoang MD, Saha A, McMillan B. Chronic odontogenic nasal discharge: report of two cases. Aust Dent J. (2021) 66(2):201-204. doi: 10.1111/adj.12789

34. Dokania V, Kinnera H, Shweta S, Shetty N, Gaikwad N. Ectopic Deciduous Maxillary Tooth in the Nasal Cavity Following Trauma. Cureus. (2021) 13(3):e14154. doi: 10.7759/cureus.14154

35. Basu S, Sahu S, Bose A, Ghosh S. Ectopic Nasal Tooth Associated with Cleft Lip and Palate. Indian J Dent Sci. (2021) 13(1):43-45. doi: 10.4103/ijds.ijds_120_19

36. Alfayez N, AlRashed ALHumaid S, Alfayez A. Ectopic nasal tooth: A case report. Int J Surg Case Rep. (2021) 88:106459. doi: 10.1016/j.ijscr.2021.106459

37. Khanna S, Turner M. Ectopic Tooth in the Nose. N Engl J Med. (2021) 385(23):e82. doi: 10.1056/NEJMicm2112363

38. Alhashim FY, Almarhoon FS, Alhashim HY, Moumen A. Endonasal endoscopic management of different cases of dentigerous cysts and ectopic teeth. J Surg Case Rep. (2021) 2021(4):rjab099. doi: 10.1093/jscr/rjab099

39. Levin M, Banglawala SM, Sommer DD. Endoscopic removal of sinonasal teeth: A case series. Oper Tech Otolaryngol Head Neck Surg. (2021) 32(2):e18-e26. doi: 10.1016/j.otot.2021.03.001

40. Gupta S, Cipolla M. Endoscopic Surgical Excision of Ectopic Tooth in Left Nasal Cavity. Cureus. (2021) 13(8):e17465. doi: 10.7759/cureus.17465

41. Ahmed T, Ahmed S, Kaushal N. Extraction of Supernumerary Nasal Tooth by Endoscopic Approach. Contemp Clin Dent. (2021) 12(3):321-323. doi: 10.4103/ccd.ccd_778_20

42. Choi YS, Kim YD, Bae CH, Na HG. Intranasal supernumerary tooth in a child: a case report. Turk J Pediatr. (2021) 63(4):731-734. doi: 10.24953/turkjped.2021.04.024

43. Ahmed AK, Alansari H, Almannai A, Ali HY. Should we floss our nose? A case report of a tooth in the nasal cavity. Int J Surg Case Rep. (2021) 80:105664. doi: 10.1016/j.ijscr.2021.105664

44. Mathur S, Verma B, Dabholkar Y, Saberwal A. Supernumerary tooth in the nasal cavity. J Oral Maxillofac Pathol. (2021) 25(2):373-374. doi: 10.4103/0973-029X.325262

45. Kilic K, Sakat MS, Sumbullu MA, Ucuncu H. Traumatic Displacement of Maxillary Teeth into the Nasal Cavity: A Diagnosis Delayed for 15 Years. West Indian Med J. (2021) 69(3):171-173. doi: 10.7727/wimj.2015.253

46. Song NY, Liu Y, He S, Liu F. A rare intranasal ectopic tooth in a child. Br J Hosp Med (Lond). (2020) 81(8):1. doi: 10.12968/hmed.2020.0230

47. Kumar V, Bhaskar A, Kapoor R, Malik P. Conservative surgical management of a supernumerary tooth in the nasal cavity. BMJ Case Rep. (2020) 13(7):e235718. doi: 10.1136/bcr-2020-235718

48. Ababtain R, Alhuthaili K, Arafat A, Aloqaili A. Ectopic intranasal tooth causing recurrent epistaxis in an adolescent patient. J Pediatr Surg Case Rep. (2020) 52:101348. doi: 10.1016/j.epsc.2019.101348

49. Kratz B, Chhabra N. Ectopic Tooth of the Nasal Cavity. Ear Nose Throat J. (2020) 99(2):NP13-NP14. doi: 10.1177/0145561318823640

50. Min HJ, Oh SR, Kim KS. Endoscopic Differences Between Intranasal Ectopic Teeth. Ear Nose Throat J. (2020) 99(6):NP58-NP59. doi: 10.1177/0145561319853511

51. Accorona R, Colombo G, Ferrari M, Fazio E, Bolzoni-Villaret A. Inverted Supernumerary Intranasal Teeth as Unusual Indications of Endoscopic Surgery. Iran J Otorhinolaryngol. (2020) 32(110):181-186. doi: 10.22038/ijorl.2019.38918.2284

52. Ramaraj PN, Mahabaleshwara CH, Singh R, George A, Vijayalakshmi G. Missing teeth after maxillofacial trauma: a case report and management protocol. J Korean Assoc Oral Maxillofac Surg. (2020) 46(6):422- 427. doi: 10.5125/jkaoms.2020.46.6.422

53. Mathew M, Sasikumar P, Vivek VJ, Kala R. Surgical management of a rare case of inverted intranasal mesiodens. BMJ Case Rep. (2020) 13(10):e237982. doi: 10.1136/bcr-2020-237982

54. Nosaka H, Yanagihara T, Oue A, Nagai M, Komori M. A case of a child with a reverse tooth in the nasal cavity treated with endoscopic removal. Oto-Rhino-Laryngology Tokyo. (2019) 62(4):175-178. Japanese. doi: 10.11453/orltokyo.62.4_175

55. Gormley M, Chahal R, Gallacher N, Bell C. A conventional surgical approach for removal of an ectopic tooth in the nasal cavity. BMJ Case Rep. (2019) 12(8):e231279. doi: 10.1136/bcr-2019-231279

56. Indeewar H, Dutt SN. Endoscopic Removal of Intranasal Supernumerary Tooth: A Case Report. Indian J Otolaryngol Head Neck Surg. (2019) 71(Suppl 3):2036-2038. doi: 10.1007/s12070-018-1455-8

57. Bergamaschi IP, Olsson B, Sebastiani AM, Trento GDS, Rebellato NLB, Klüppel LE, et al. Intranasal Ectopic Tooth in Adult and Pediatric Patients: A Report of Two Cases. Case Rep Surg. (2019) 2019:8351825. doi: 10.1155/2019/8351825

58. Jones L, Hilton JM, Heyman J, Viswanathan H, Wreesmann VB, Hayes SM. Nasal obstruction secondary to an inverted midline supernumerary tooth. Ann R Coll Surg Engl. (2019) 101(1):e14-e16. doi: 10.1308/rcsann.2018.0163

59. Biswas S, Sarkar S, Bandyopadhyay D, Patra C. Recurrent Unilateral Nasal Obstruction: A Cause Lying Deep Inside. J Nepal Paediatr Soc. (2019) 39(2):123-126. doi: 10.3126/jnps.v39i2.26911

60. Moeller ML, Bille J, Fuglsang M. Retained tooth in the nasal cavity: a rare cause of nasal congestion. BMJ Case Rep. (2019) 12(2):e226745. doi: 10.1136/bcr-2018-226745

61. Costa SM, de Jesus AO, Silveira RL, Amaral MBF. Supernumerary nasal tooth removed with a modified maxillary vestibular approach: case report and literature review. Oral Maxillofac Surg. (2019) 23(2):247-252. doi: 10.1007/s10006-019-00763-4

62. Jin KF, Lou ZC, Lou ZH. The diagnosis and management of supernumerary nasal teeth. J Laryngol Otol. (2019) 133(6):538-541. doi: 10.1017/S0022215119000793

63. Albert A, Mupparapu M. Cone beam computed tomography review and classification of mesiodens: Report of a case in the nasal fossa and nasal septum. Quintessence Int. (2018) 49(5):413-417. doi: 10.3290/j.qi.a40052

64. Koçak HE, Özdamar K, Bilgi B, Acıpayam H. A Rare Cause of Intranasal Mass: Bilateral Ectopic Nasal Teeth. Iran J Otorhinolaryngol. (2017) 29(94):287-289. doi: 10.22038/ijorl.2016.15277.1543

65. Ogane S, Watanabe A, Takano N, Shibahara T. Case of Inverted Supernumerary Tooth in Nasal Cavity. Bull Tokyo Dent Coll. (2017) 58(4):255-258. doi: 10.2209/tdcpublication.2016-0040

66. Cruz Toro P, Clemente I, Domènech I. Intranasal ectopic tooth. Acta Otorrinolaringol Esp (Engl Ed). (2017) 68(5):305-306. doi: 10.1016/j.otorri.2016.07.005

67. Hägerström E, Nepper-Christensen S. [Ectopic tooth as a rare cause of foreign body reaction of the nose]. Ugeskr Laeger. (2016) 178(21):V12150956. Danish.

68. Wang XY, Tian JS, Lu ZB, Bai YD, Zhang HQ. [Maxillary supernumerary teeth are misdiagnosed as nasal foreign body: one case]. Lin Chuang Er Bi Yan Hou Tou Jing Wai Ke Za Zhi. (2016) 30(22):1811-1812. Chinese. doi: 10.13201/j.issn.1001-1781.2016.22.019

69. Li S, Wu J. [Supernumerary nasal tooth a case report]. Lin Chuang Er Bi Yan Hou Tou Jing Wai Ke Za Zhi. (2016) 30(1):78-9. Chinese.

70. Acharya S, Mishra UP. Intranasal ectopic tooth presenting as rhinolith - a rare case report. J Evol Med Dent Sci. (2016) 5(85):6350-6351. doi: 10.14260/jemds/2016/1434

71. Guo G, Hu L, Lu Y. [Nasal cavity supernumerary tooth and maxillary sinusitis: one case reported]. Lin Chuang Er Bi Yan Hou Tou Jing Wai Ke Za Zhi. (2015) 29(16):1494-5. Chinese.

72. Yu C, Gu D, An J, Tang Y. Case presentation of an intranasal ectopic tooth in a pediatric patient. Am J Otolaryngol. (2015) 36(3):472-4. doi: 10.1016/j.amjoto.2015.01.020

73. Kim KS, Min HJ, Yang HS. Fully Erupted Intranasal Tooth Mimicking Benign Tumor. J Craniofac Surg. (2015) 26(6):e556-7. doi: 10.1097/SCS.0000000000002013

74. Lai WS, Lee JC. Intranasal ectopic tooth. Indian J Med Res. (2014) 140(5):696.

75. Agrawal M, Khan TS, Gupta T, Khanna S. Intranasal tooth: ectopic eruption 1 year after maxillofacial trauma. BMJ Case Rep. (2014) 2014:bcr2014204432. doi: 10.1136/bcr-2014-204432

76. Al Dhafeeri HO, Kavarodi A, Al Shaikh K, Bukhari A, Al Hussain O, El Baramawy A. Recurrent epistaxis caused by an intranasal supernumerary tooth in a young adult. Am J Case Rep. (2014) 15:291-3. doi: 10.12659/AJCR.890710

77. Tang S, Reisacher W. Supernumerary nasal tooth in close proximity to a dental implant. J Oral Maxillofac Surg. (2014) 72(12):2420.e1-4. doi: 10.1016/j.joms.2014.08.031

78. Yang Y, Duan X, Chen Y. [Familial nasal bottom supernumerary tooth dentigerous cyst: report of 1 case and review of literatures]. Lin Chuang Er Bi Yan Hou Tou Jing Wai Ke Za Zhi. (2013) 27(20):1158-9. Chinese.

79. Krishnan B, Parida PK, Gopalakrishnan S, Satyparakash MV. An unusual cause of epistaxis in a young patient: the supernumerary nasal tooth. Oral Maxillofac Surg. (2013) 17(4):315-7. doi: 10.1007/s10006-013-0393-6

80. Mohebbi S, Salehi O, Ebrahimpoor S. Ectopic supernumerary tooth in nasal septum: a case study. Iran J Otorhinolaryngol. (2013) 25(72):183-6.

81. Noleto JW, Prado R, Rocha JF, DaCosta MA, Barbosa CU, Toscano MD. Intranasal inverted tooth: a rare cause of a persistent rhinosinusitis. Indian J Dent Res. (2013) 24(6):762-4. doi: 10.4103/0970-9290.127630

82. van Essen TA, van Rijswijk JB. 'Intranasal toothache': case report. J Laryngol Otol. (2013) 127(3):321-2. doi: 10.1017/S0022215112002927

83. Janardhan N, Kumar SR, Reddy RR, Kumar CA. Rhinolithiasis due to supernumerary ectopic tooth: very rare case. Indian J Otolaryngol Head Neck Surg. (2013) 65(4):383-4. doi: 10.1007/s12070-012-0493-x

84. Nathan K, Nagala V, Farhat S, Shah A. An unusual foreign body in the nostril. J Laryngol Otol. (2012) 126(9):952-4. doi: 10.1017/S0022215112001351

85. Zalagh M, Akhaddar A, Benariba F. Chronic rhinorrhea revealing an actinomycotic rhinolithiasis with ectopic tooth. Int J Oral Maxillofac Surg. (2012) 41(3):297-9. doi: 10.1016/j.ijom.2011.07.901

86. Verma RK, Bakshi J, Panda NK. Ectopic intranasal tooth: an unusual cause of epistaxis in a child. Ear Nose Throat J. (2012) 91(6):242-4. doi: 10.1177/014556131209100606

87. Iwai T, Aoki N, Yamashita Y, Omura S, Matsui Y, Maegawa J, et al. Endoscopic removal of bilateral supernumerary intranasal teeth. J Oral Maxillofac Surg. (2012) 70(5):1030-4. doi: 10.1016/j.joms.2011.10.014

88. Ricks RG, Shaaban A. Nasal tooth: an unusual cause of nasal obstruction and sinusitis. BMJ Case Rep. (2012) 2012:bcr0920114767. doi: 10.1136/bcr.09.2011.4767

89. Sammartino G, Trosino O, Perillo L, Cioffi A, Marenzi G, Mortellaro C. Alternative transoral approach for intranasal tooth extraction. J Craniofac Surg. (2011) 22(5):1944-6. doi: 10.1097/SCS.0b013e31821151ba

90. Uppal SK, Gupta A, Rai R, Nippun N. Incisor tooth in the nose: anecdotal sequel to dog bite in a 3-year-old child. Indian J Dent Res. (2011) 22(4):606-7. doi: 10.4103/0970-9290.90313

91. Prasad Guru R, Nair PP, Gharote H, Hegade K, Agarwal K, Jain A. Intranasal Tooth—An Ectopic Eruption of Mesiodens in Nasal Cavity: A Case Report and Review. J Indian Acad Oral Med Radiol. (2011) 23:252-255. doi: 10.5005/jp-journals-10011-1140

92. Chrcanovic BR, Bueno SC, da Silveira DT, Custódio AL. Traumatic displacement of maxillary permanent incisor into the nasal cavity. Oral Maxillofac Surg. (2010) 14(3):175-82. doi: 10.1007/s10006-009-0191-3

93. Sanei-Moghaddam A, Hyde N, Williamson P. Endoscopic removal of a supernumerary tooth from the nasal cavity in an adult. Br J Oral Maxillofac Surg. (2009) 47(6):484-5. doi: 10.1016/j.bjoms.2009.04.036

94. Kirmeier R, Truschnegg A, Payer M, Malyk J, Daghighi S, Jakse N. The supernumerary nasal tooth. Int J Oral Maxillofac Surg. (2009) 38(11):1219-22. doi: 10.1016/j.ijom.2009.06.013

95. Choudhury B, Das AK. Supernumerary Tooth in the Nasal Cavity. Med J Armed Forces India. (2008) 64(2):173-4. doi: 10.1016/S0377-1237(08)80071-8

96. Luna AH, Moreira RW, de Moraes M. Traumatic intrusion of maxillary permanent incisors into the nasal cavity: report of a case. Dent Traumatol. (2008) 24(2):244-7. doi: 10.1111/j.1600-9657.2007.00524.x

97. Arunkumar JS, Prasad KC, Shanthi N. Nasal teeth: A case report. Indian J Otolaryngol Head Neck Surg. (2007) 59(2):197-8. doi: 10.1007/s12070-007-0060-z

98. Shaw LCK. Rhinolith of endogenous origin: A rare entity. Surg Pract. (2007) 11(1):48-50. doi: 10.1111/j.1744-1633.2007.00329.x

99. Öztürk C, Eryilmaz K, Çakur B. Supernumerary tooth in the nose. Turk J Med Sci. (2007) 37(4):227-230.

100. Lee JH. A nasal tooth associated with septal perforation: A rare occurrence. Eur Arch Otorhinolaryngol. (2006) 263(11):1055-6. doi: 10.1007/s00405-006-0107-x

101. Senkal HA, Suslu AE, Unal OF. A rare cause of rhinolithiasis: Ectopic tooth. Int J Pediatr Otorhinolaryngol Extra. (2006) 1(3):249-252. doi: 10.1016/j.pedex.2006.07.004

102. Jones HB, Whitley SP, Morelli G, Tuffin JR. Interesting case: Spontaneous exfoliation of an upper central incisor through the nasal floor. Br J Oral Maxillofac Surg. (2006) 44(6):500. doi: 10.1016/j.bjoms.2006.03.011

103. Kakade A, Gandhy MM, Damle SG. Management of nasally erupting deciduous canine in operated cleft lip and alveolus patient. J Indian Soc Pedod Prev Dent. (2006) 24(1):40-1. doi: 10.4103/0970-4388.22834

104. Gilbride MJ, Smith WP. Eruption of teeth in the nose following trauma to the primary and permanent dentitions. Br Dent J. (2005) 198(4):199-200. doi: 10.1038/sj.bdj.4812081

105. Eichhorn KW, Wardelmann E, Bücheler M. [Chronic nose blockade and rhinorrhea]. HNO. (2004) 52(10):908-10. German. doi: 10.1007/s00106-004-1100-6

106. Wang LF, Tai CF, Lee KW, Ho KY, Kuo WR. Delayed removal of a fully intruded tooth in the nasal cavity after facial trauma: a case report. Otolaryngol Head Neck Surg. (2004) 131(3):330-1. doi: 10.1016/j.otohns.2003.08.026

107. Lin IH, Hwang CF, Su CY, Kao YF, Peng JP. Intranasal tooth: report of three cases. Chang Gung Med J. (2004) 27(5):385-9.

108. Kuroda H, Tsutsumi K, Tomisawa H, Koizuka I. A case of an inverted tooth in the nasal cavity. Auris Nasus Larynx. (2003) 30 Suppl:S127-9. doi: 10.1016/s0385-8146(02)00131-1

109. Aoki M, Jinbu Y, Matsumura T, Kusama M. Ectopic Tooth in the Nasal Cavity. Asian J Oral Maxillofac Surg. (2003) 15(3):218-220. doi: 10.1016/s0915-6992(03)80047-x

110. Kim DH, Kim JM, Chae SW, Hwang SJ, Lee SH, Lee HM. Endoscopic removal of an intranasal ectopic tooth. Int J Pediatr Otorhinolaryngol. (2003) 67(1):79-81. doi: 10.1016/s0165-5876(02)00290-2

111. Chu CK, Chiang CW. Intranasal Tooth - A Case Report. Tzu Chi Med J. (2003) 15(6):421-424.

112. Bhagat DR, Singh P, Kotwal S, Sharma A, Mahotra J. Nasal tooth. Indian J Otolaryngol Head Neck Surg. (2003) 55(4):282-4. doi: 10.1007/BF02992440

113. Martin BS. Traumatic intrusion of maxillary permanent incisors into the nasal cavity associated with a seizure disorder: report of a case. Dent Traumatol. (2003) 19(5):286-8. doi: 10.1034/j.1600-9657.2003.00170.x

114. Thor AL. Delayed removal of a fully intruded primary incisor through the nasal cavity: a case report. Dent Traumatol. (2002) 18(4):227-30. doi: 10.1034/j.1600-9657.2002.00070.x

115. Chen A, Huang JK, Cheng SJ, Sheu CY. Nasal teeth: report of three cases. AJNR Am J Neuroradiol. (2002) 23(4):671-3.

116. Lee FP. Endoscopic extraction of an intranasal tooth: a review of 13 Cases. Laryngoscope. (2001) 111(6):1027-31. doi: 10.1097/00005537-200106000-00017

117. Gupta YK, Shah N. Intranasal tooth as a complication of cleft lip and alveolus in a four year old child: case report and literature review. Int J Paediatr Dent. (2001) 11(3):221-4. doi: 10.1046/j.1365-263x.2001.00251.x

118. Moreano EH, Zich DK, Goree JC, Graham SM. Nasal tooth. Am J Otolaryngol. (1998) 19(2):124-6. doi: 10.1016/s0196-0709(98)90108-9

119. Tung TC, Chen YR, Chen CT, Lin CJ. Full intrusion of a tooth after facial trauma. J Trauma. (1997) 43(2):357-9. doi: 10.1097/00005373-199708000-00026

120. Chamyal PC. Intranasal supernumerary tooth. Indian J Otolaryngol Head Neck Surg. (1997) 49(1):54-6. doi: 10.1007/BF02991715

121. Cobourne MT, Bloom KL, Harley KE, Porter SR. An unusual dental cause of nasal discharge: a case report. Int J Paediatr Dent. (1996) 6(3):187-9. doi: 10.1111/j.1365-263x.1996.tb00240.x

122. Nastri AL, Smith AC. The nasal tooth. Case report. Aust Dent J. (1996) 41(3):176-7. doi: 10.1111/j.1834-7819.1996.tb04851.x

123. Pracy JP, Williams HO, Montgomery PQ. Nasal teeth. J Laryngol Otol. (1992) 106(4):366-7. doi: 10.1017/s0022215100119516

124. Ohashi N, Hori M. A case report of supernumerary tooth and review of literature. Auris Nasus Larynx. (1991) 18(2):183-7. doi: 10.1016/s0385-8146(12)80223-9

125. Carver DD, Peterson S, Owens T. Intranasal teeth: a case report. Oral Surg Oral Med Oral Pathol. (1990) 70(6):804-5. doi: 10.1016/0030-4220(90)90026-o

126. Ogisi FO, Odita JC. Ectopic nasal dentition associated with squamous cell carcinoma of palate in a 12-year-old boy. Br J Oral Maxillofac Surg. (1988) 26(1):58-61. doi: 10.1016/0266-4356(88)90151-9

127. King NM, Lee AM. An intranasal tooth in a patient with a cleft lip and palate: report of case. J Am Dent Assoc. (1987) 114(4):475-8. doi: 10.14219/jada.archive.1987.0124

128. Spencer MG, Couldery AD. Nasal tooth. J Laryngol Otol. (1985) 99(11):1147-50. doi: 10.1017/s0022215100098327

129. Johnson AP. A case of an intranasal canine tooth. J Laryngol Otol. (1981) 95(12):1277-9. doi: 10.1017/s0022215100092148

130. Dayal PK, Dewan SK, Bihani VK, Dave CJ. Eruption of a tooth into the nasal cavity due to osteomyelitis. J Laryngol Otol. (1981) 95(5):509-12. doi: 10.1017/s0022215100091027

131. Smith RA, Gordon NC, De Luchi SF. Intranasal Teeth. Report of two cases and review of the literature. Oral Surg Oral Med Oral Pathol. (1979) 47(2):120-2. doi: 10.1016/0030-4220(79)90163-4

132. Thawley SE, LaFerriere KA. Supernumerary nasal tooth. Laryngoscope. (1977) 87(10 Pt 1):1770-3. doi: 10.1288/00005537-197710000-00025

133. Hillis RE. An impacted supernumerary tooth in the floor of the right naris. Report of a case. Oral Surg Oral Med Oral Pathol. (1972) 33(6):894-8. doi: 10.1016/0030-4220(72)90179-x

134. Martinson FD, Cockshott WP. Ectopic nasal dentition. Clin Radiol. (1972) 23(4):451-4. doi: 10.1016/s0009-9260(72)80015-1

135. Lumba SP, Nirola A, Grewal BS. Healed osteomyeliis of Maxilla with tooth in the floor of nose. J Laryngol Otol. (1971) 85(8):877-9. doi: 10.1017/s002221510007417x

136. Kohli GS, Verma PL. Ectopic supernumerary tooth in the nasal cavity. J Laryngol Otol. (1970) 84(5):537-8. doi: 10.1017/s0022215100072200

137. Rege SR, Shah KL, Marfatia PT. Osteomyelitis of maxilla with extrusion of teeth in the floor of the nose requiring extraction. J Laryngol Otol. (1970) 84(5):533-5. doi: 10.1017/s0022215100072194

138. Chopra SS, Joshi MR. Mesiodens erupted in the nasal cavity. Report of a case. Oral Surg Oral Med Oral Pathol. (1969) 28(6):856-8. doi: 10.1016/0030-4220(69)90338-7

139. Hiranandani LH, Melgiri RD. Supernumerary tooth in the floor of the nose. J Laryngol Otol. (1968) 82(9):845-8. doi: 10.1017/s0022215100069565

# Reference number mapping

| **Ref no.** | **NT identifier** | **Ref no.** | **NT identifier** | **Ref no.** | **NT identifier** |
| --- | --- | --- | --- | --- | --- |
| 1 | NT-001 | 48 | NT-059 | 95 | NT-132 |
| 2 | NT-002 | 49 | NT-060 | 96 | NT-134 |
| 3 | NT-003 | 50 | NT-061 | 97 | NT-137 |
| 4 | NT-004 | 51 | NT-062 28 | 98 | NT-138 |
| 5 | NT-005 | 52 | NT-063 | 99 | NT-139 |
| 6 | NT-006 | 53 | NT-064 | 100 | NT-140 |
| 7 | NT-007 | 54 | NT-065 | 101 | NT-141 |
| 8 | NT-008 | 55 | NT-067 30 | 102 | NT-143 |
| 9 | NT-010 | 56 | NT-070 | 103 | NT-144 |
| 10 | NT-011 | 57 | NT-073 | 104 | NT-147 |
| 11 | NT-012 | 58 | NT-074 | 105 | NT-150 |
| 12 | NT-013 | 59 | NT-075 | 106 | NT-151 |
| 13 | NT-014 | 60 | NT-076 | 107 | NT-152 25 |
| 14 | NT-015 | 61 | NT-077 | 108 | NT-156 |
| 15 | NT-016 | 62 | NT-078 | 109 | NT-158 |
| 16 | NT-018 31 | 63 | NT-079 27 | 110 | NT-159 |
| 17 | NT-019 | 64 | NT-081 | 111 | NT-161 |
| 18 | NT-021 | 65 | NT-082 | 112 | NT-162 |
| 19 | NT-023 | 66 | NT-083 | 113 | NT-163 |
| 20 | NT-024 | 67 | NT-084 | 114 | NT-165 |
| 21 | NT-026 | 68 | NT-085 | 115 | NT-166 |
| 22 | NT-027 | 69 | NT-086 | 116 | NT-167 20 |
| 23 | NT-028 | 70 | NT-088 | 117 | NT-168 |
| 24 | NT-219 | 71 | NT-090 | 118 | NT-175 |
| 25 | NT-030 | 72 | NT-092 | 119 | NT-178 |
| 26 | NT-032 | 73 | NT-095 | 120 | NT-179 |
| 27 | NT-033 | 74 | NT-099 | 121 | NT-180 |
| 28 | NT-035 | 75 | NT-100 | 122 | NT-185 |
| 29 | NT-036 | 76 | NT-101 26 | 123 | NT-191 |
| 30 | NT-040 | 77 | NT-102 | 124 | NT-192 |
| 31 | NT-041 | 78 | NT-104 | 125 | NT-195 |
| 32 | NT-042 | 79 | NT-105 | 126 | NT-202 |
| 33 | NT-043 | 80 | NT-106 24 | 127 | NT-205 |
| 34 | NT-044 17 | 81 | NT-107 | 128 | NT-206 |
| 35 | NT-045 | 82 | NT-109 | 129 | NT-210 |
| 36 | NT-046 | 83 | NT-110 | 130 | NT-211 |
| 37 | NT-047 | 84 | NT-115 | 131 | NT-214 |
| 38 | NT-048 | 85 | NT-116 | 132 | NT-218 |
| 39 | NT-049 | 86 | NT-117 | 133 | NT-226 |
| 40 | NT-050 | 87 | NT-118 | 134 | NT-227 |
| 41 | NT-051 | 88 | NT-120 | 135 | NT-229 |
| 42 | NT-052 | 89 | NT-122 | 136 | NT-230 |
| 43 | NT-053 | 90 | NT-123 | 137 | NT-231 |
| 44 | NT-054 | 91 | NT-124 | 138 | NT-234 |
| 45 | NT-055 | 92 | NT-126 18 | 139 | NT-236 |
| 46 | NT-056 | 93 | NT-127 |  |  |
| 47 | NT-058 | 94 | NT-129 |  |  |
